# Cellular Therapy Using Epitope-Imprinted Composite Nanoparticles to Remove α-Synuclein from an In Vitro Model

**DOI:** 10.3390/cells11162584

**Published:** 2022-08-19

**Authors:** Mei-Hwa Lee, Jeng-Shiung Jan, James L. Thomas, Yuan-Pin Shih, Jin-An Li, Chien-Yu Lin, Tooru Ooya, Lilla Barna, Mária Mészáros, András Harazin, Gergő Porkoláb, Szilvia Veszelka, Maria A. Deli, Hung-Yin Lin

**Affiliations:** 1 Department of Materials Science and Engineering, I-Shou University, Kaohsiung 84001, Taiwan; 2 Department of Chemical Engineering, National Cheng Kung University, Tainan 70101, Taiwan; 3 Department of Physics and Astronomy, University of New Mexico, Albuquerque, NM 87131, USA; 4 Department of Chemical and Materials Engineering, National University of Kaohsiung, Kaohsiung 81148, Taiwan; 5 Graduate School of Engineering, Department of Chemical Science and Engineering, Kobe University, Kobe 657-8501, Japan; 6 Center for Advanced Medical Engineering Research & Development (CAMED), Kobe University, Kobe 657-8501, Japan; 7 Institute of Biophysics, Biological Research Centre, H-6726 Szeged, Hungary; 8 Doctoral School in Biology, University of Szeged, H-6720 Szeged, Hungary

**Keywords:** α-synuclein, peptide imprinting, magnetic nanoparticles, gene activation, protein extraction

## Abstract

Several degenerative disorders of the central nervous system, including Parkinson’s disease (PD), are related to the pathological aggregation of proteins. Antibodies against toxic disease proteins, such as α-synuclein (SNCA), are therefore being developed as possible therapeutics. In this work, one peptide (YVGSKTKEGVVHGVA) from SNCA was used as the epitope to construct magnetic molecularly imprinted composite nanoparticles (MMIPs). These composite nanoparticles were characterized by dynamic light scattering (DLS), high-performance liquid chromatography (HPLC), isothermal titration calorimetry (ITC), Brunauer–Emmett–Teller (BET) analysis, and superconducting quantum interference device (SQUID) analysis. Finally, the viability of brain endothelial cells that were treated with MMIPs was measured, and the extraction of SNCA from CRISPR/dCas9a-activated HEK293T cells from the in vitro model system was demonstrated for the therapeutic application of MMIPs.

## 1. Introduction

Protein toxicity is thought to be implicated in many neurodegenerative diseases, including Alzheimer’s disease, Parkinson’s disease (PD), amyotrophic lateral sclerosis, frontotemporal dementia, and Huntington’s disease [1]; in fact, protein toxicity is the cardinal feature of these neurodegenerative diseases [2]. The fastest-growing treatment strategy for neurodegenerative diseases is the utilization of antibodies [3] to eliminate toxic proteins. Accordingly, antibodies against toxic disease proteins, such as α-synuclein (SNCA) and β-amyloid, are being developed [1].

Lewy bodies are intraneuronal aggregates of SNCA protein that are characteristic of PD, Lewy body dementia, and other disorders [4]. They are found in the regions of the brain that are responsible for motor control, and cause a progressive decline in mental ability [5]. Duplications and triplications of SNCA have been implicated in PD; even a 1.5–2-fold increase in SNCA expression may contribute to PD [6]. Furthermore, the levels of steady-state SNCA mRNA are sporadic in both PD brain samples and normal controls from mid-brain tissue, including the substantia nigra [7]. SNCA mRNA levels in PD brains have been found to be an average of nearly fourfold higher than those in control mid-brain tissue, although the variability in samples from PD patients is much greater than that in control samples [7]. 

Molecularly imprinted polymers (MIPs) have been synthesized for the recognition of target molecules, and have been widely used in bioseparations [8,9,10], biosensing [8,11,12,13,14], and medicines [15,16]. Zhang’s group developed epitope-imprinted nanoparticles for the recognition of oxytocin [17], bovine serum albumin [18,19], tyrosine phosphopeptide [20], human serum albumin [21], and cytochrome c [22,23,24]. Peptides of CRISPR (clustered regularly interspaced short palindromic repeat)-associated protein 9 have also been used as templates for MIPs to extract Cas9 proteins from transfected cells, and to deliver genes for cellular reprograming [25]. Peptide-imprinted conducting polymers have been used to increase signal amplification during the electrochemical sensing of SNCA [26,27,28]. In vitro studies based on increased expression of SNCA constitute an important means for studying the molecular basis of synucleinopathies [29]. A synthetic antibody for SNCA may be able to remove SNCA proteins from cells and/or the intercellular medium, thus, in principle, providing therapeutic benefits similar to those achievable with other antibodies, but at lower cost [3]. In the present work, one peptide from the sequence of SNCA was used as a template for the synthesis of magnetic molecularly imprinted polymer nanoparticles (MMIPs), and, as proof-of-principle, used to extract SNCA from cell culture medium.

## 2. Materials and Methods

**Scheme 1:**
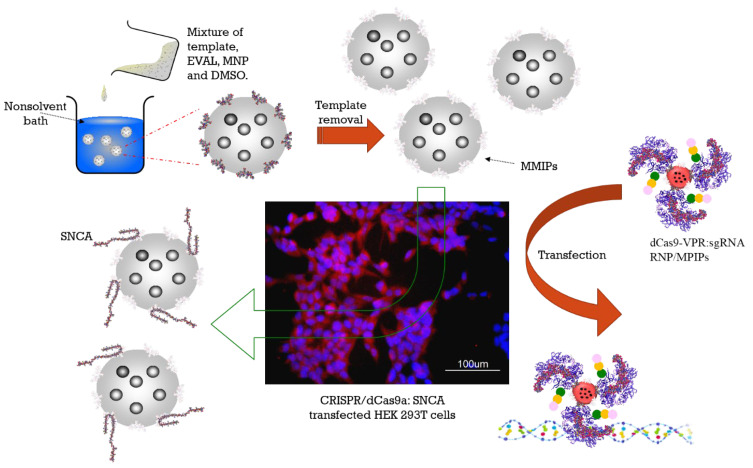
The preparation of magnetic molecularly imprinted nanoparticles (MMIPs) and their use in the extraction of SNCA from CRISPR/dCas9-activated HEK293T cells.

Poly(ethylene-*co*-vinyl alcohol), EVAL, was used as the imprinted polymer. Magnetic nanoparticles were encapsulated within the MIP particles during their formation to aid in particle manipulation and separations. The size distributions of MMIPs, comprising various EVAL compositions and imprinting peptide concentrations, were measured by dynamic light scattering (DLS). To optimize the preparation conditions, the peptide binding capacities of the MMIPs were compared using high-performance liquid chromatography (HPLC) and isothermal titration calorimetry (ITC). The specific surface areas and magnetization properties were obtained by Brunauer–Emmett–Teller (BET) and superconducting quantum interference device (SQUID) analyses, respectively. Finally, the cytotoxic index for brain endothelial cells, and the extraction of SNCA from CRISPR/dCas9a-activated HEK293T cells were both examined. Scheme 1 shows the preparation of magnetic molecularly imprinted nanoparticles (MMIPs) and their use for the extraction of SNCA from the CRISPR/dCas9-activated HEK293T cells. The wildtype sequence of peptide P5 ^39^YVGSKTKEGVVHGVA^53^ that contains the KTKEGV consensus sequence was selected for imprinting. This consensus sequence is thought to play a role in multimerization and aggregate formation. The experimental subsection of the Materials and Methods can be found in the electronic Supplementary Materials (ESM) Files S1 and S2.

## 3. Results and Discussion

**Figure 1:**
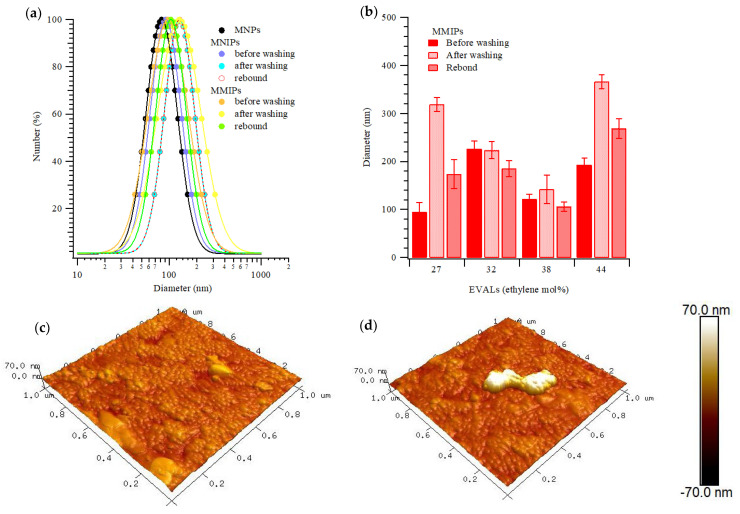
(**a**) DLS size distributions of magnetic non- and peptide-imprinted composite nanoparticles before and after peptide removal and rebinding. (**b**) Mean sizes of nanoparticles prepared with EVALs with various ethylene mol%. AFM images of magnetic (**c**) non- and (**d**) peptide-imprinted composite nanoparticles after peptide removal.

Figure 1 presents the size distribution and morphologies of magnetic non-imprinted (MNIPs) and peptide-imprinted (MMIPs) polymeric particles as measured using a DLS sizer and AFM. The MMIPs were prepared using 38 mol% ethylene EVAL and peptide P5 as the template at a concentration of 0.1 wt%. The magnetic nanoparticles (MNPs) have a mean diameter of 90 nm with a polydispersity of 0.188. From the overall size of the MMIPs and MNIPs, the EVAL coatings on the MNIPs and MMIPs are about 90–150 nm, but caution should be used in interpreting this number, as the washing of NPs may induce their aggregation. Interestingly, the rebinding of peptide to the MMIPs reduces their size to 94 ± 20 nm. The imprinting of peptides using different ethylene mol% of EVAL resulted in particle sizes ranging from 100 to about 400 nm (Figure 1b), varying non-monotonically with ethylene content (and thus hydrophobicity). As will be discussed below, the more hydrophobic EVALs (38 and 44 mol% ethylene) exhibited the best selectivity for target peptide binding, however, there appears to be no correlation between particle size and selectivity. Figure 1c,d display AFM images of MNIPs and MMIPs, respectively. The size of the aggregated particles is consistent with the results of DLS, but many smaller MNIPs and MMIPs are also observed. 

**Figure 2:**
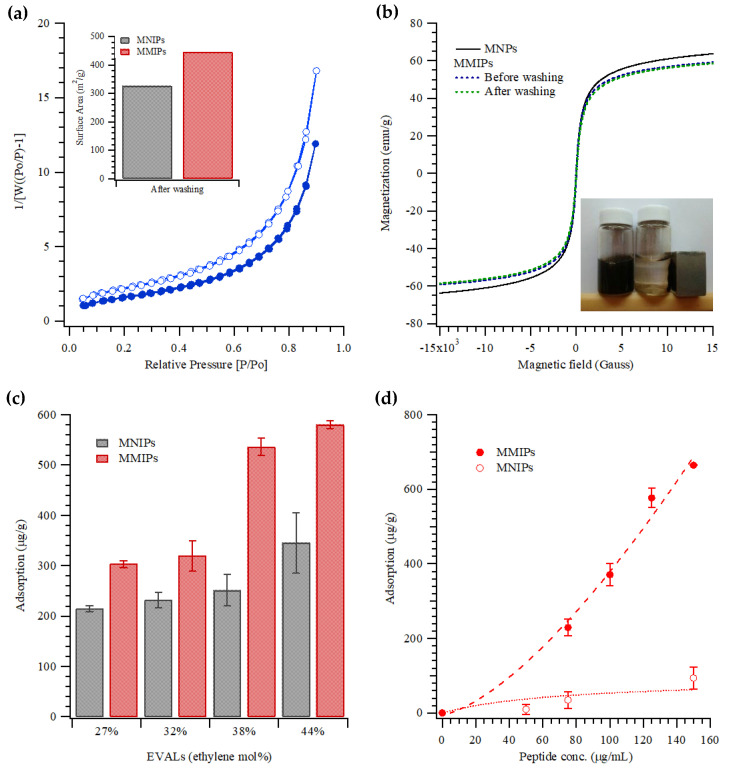
(**a**) BET analysis and specific surface areas (inset) of MNIP and MMIP nanoparticles. (**b**) SQUID magnetization measurement of MMIPs before and after template removal. MNPs are magnetic nanoparticles alone. (**c**) Peptide binding to imprinted and non-imprinted nanoparticles, showing increasing imprinting effectiveness with higher ethylene content. (**d**) Binding isotherm of MNIP and MMIP nanoparticles.

Figure 2 shows the additional characterization of MNIPs and MMIPs. The specific surface area of MMIPs may be related to the rebinding capacity; therefore, surface area was measured via adsorption and desorption of nitrogen on MNIP and MMIPs, as presented in Figure 2a (EVAL, 38 mol% ethylene). The specific surface areas of MMIPs and MNIPS are 445.5 and 326.6 m^2^/g, respectively. Figure 2b displays the magnetization of the MNPs and MMIPs (EVAL, 38 mol% ethylene) before and after template removal. The magnetic nanoparticles have a saturated magnetization of 63.7 emu/g, while the MMIPs are 32.7 emu/g when template is still bound. This increases to 59.2 emu/g after template removal. Not surprisingly, the coating of MNPs with MIPs reduces their saturated magnetization (per gram). Figure 2c presents the adsorption of peptides on MMIPs and MNIPs synthesized using EVAL with ethylene in various mole ratios. Higher ethylene content (higher hydrophobicity) was seen to increase the adsorption of peptides onto both imprinted and non-imprinted particles. However, the increase was larger for imprinted MIPs, and thus, the selectivity was also higher. Figure 2d shows that the absorption capacity of MMIPs is higher than 600 μg/g.

**Figure 3:**
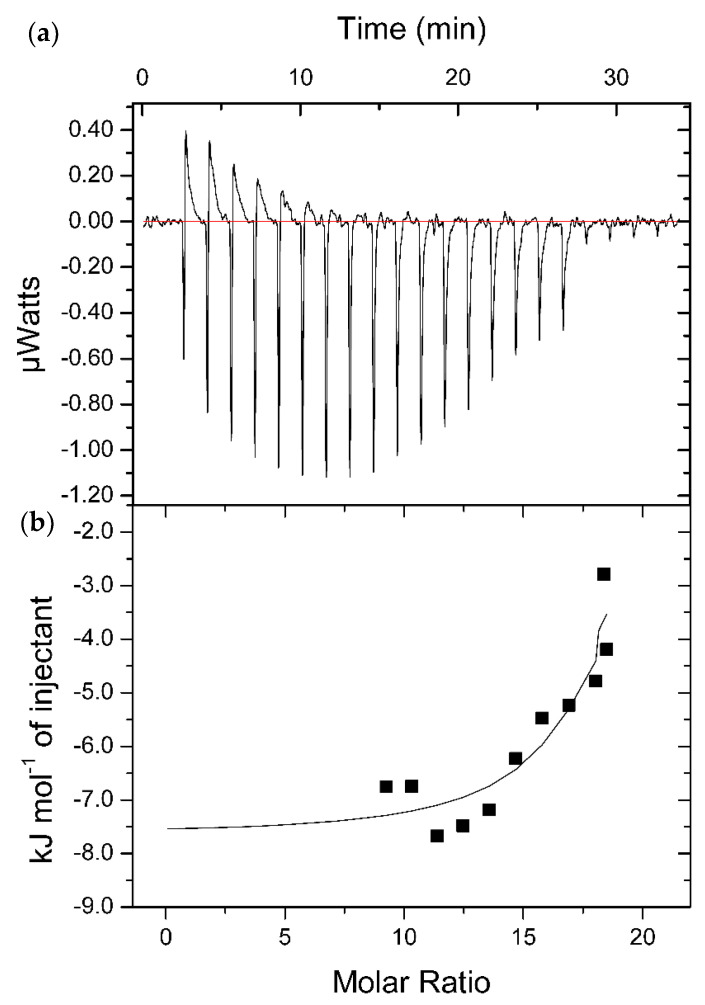
(**a**) ITC titration curves and (**b**) the corresponding fitting curve for MMIPs and P5 peptide in water. In this experiment, a suspension of MMIPs (33 μg/mL, 6.6 μM, assuming 0.198 mmol/g binding sites) was titrated with a solution of P5 peptide (1.0 mg/mL: total concentration; 0.66 mM).

In order to estimate the number of biding sites per MMIP particle, and the affinity of MMIPs for the P5 peptide, we conducted isothermal titration calorimetry (ITC). As shown in Figure 3a, upon the addition of the P5 peptide, both endothermic and exothermic heat was observed until the 8th injection. After the 9th injection, exothermic heat disappeared, and the endothermic peaks decreased. Since the MMIPs are initially aggregated (as shown in Figure 1d), the exothermic heat until the 8th injection is likely due to the dissociation of the aggregated MMIP particles in the aqueous media by the addition of the P5 peptide, and the endothermic heat arises from the binding of the P5 peptide to the MMIPs. (It is noteworthy that the weak initial aggregation does not inhibit peptide binding). To avoid conflating dissociation with binding, we carried out curve fitting from the 9th injection peak based on a basic one-site binding model, including a stoichiometry parameter (N-value) that reflects the number of peptides that can bind to each nanoparticle. In this case, the best fitting curve (Figure 3b) exhibited an N-value of 18.5 ± 0.5, suggesting that MMIPs have multiple peptide binding sites. The values obtained for the binding affinity (*K_a_*) and enthalpy of binding (Δ*H*) were 7.73 × 10^5^ (M^−1^) and −7.62 kJ/mol, respectively. 

**Figure 4:**
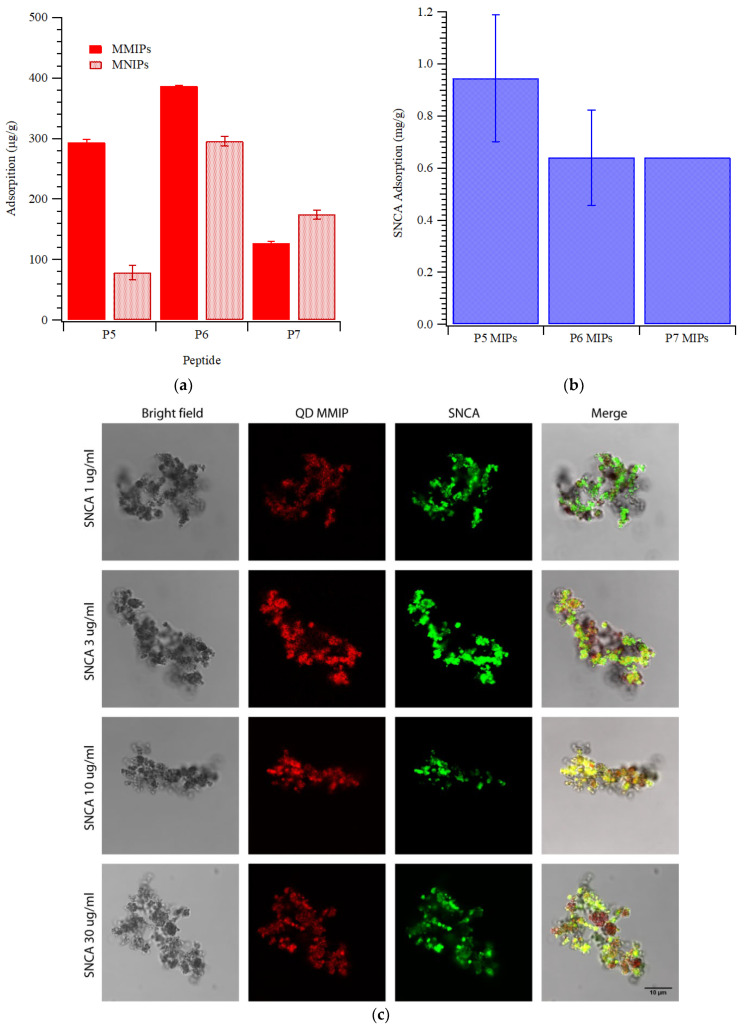
(**a**) Adsorption of P5, P6, and P7 on the P5 MMIPs and MNIPs. (**b**) Adsorption of P5, P6, and P7 on P5, P6, and P7 MMIPs, respectively. (**c**) Immunostaining for SNCA in quantum-dot-labeled SNCA recognizing magnetic nanoparticles (QD@MMIP). QD@MMIPs (100 µg/mL) were preincubated with 1, 3, 10, or 30 µg/mL SNCA for 30 min. Scale bar: 10 µm. Images are composites of z-stacks, and thus, the relative intensities are not significant.

The binding of SNCA peptides P5, P6, and P7 to P5-imprinted MMIPs and NIPs is shown in Figure 4a. (P6 differs from P5 in terms of an A53T substitution, and P7 differs in terms of A53T and E46K). For the P5 target, the imprinting effectiveness (amount bound to MMIPs divided by the amount bound to MNIPs) was nearly 4. Interestingly, however, more of peptide P6 actually bound to the P5 MMIP than did peptide P5. This binding was almost entirely non-specific, however, as evidenced by the very high level of binding to non-imprinted MNIPs. P7 binding was lower (<150 mg/g) and entirely non-specific. Figure 4b shows the adsorption of SNCA on MMIPs imprinted with either P5, P6, or P7. P5-imprinted MMIPs bind about 1.5 times as much as SNCA on MMIPs recognizing the other peptide targets. Figure S1 shows the CD spectra of P5–P7 peptides. All three peptides exhibited CD minima at 190–197 nm, followed by a maximum at 197–205 nm, suggesting that these peptides adopted mainly a random coil conformation with a low fraction of ordered conformation (that is α-helices and β-sheets, which would be evidenced by the presence of a minimum at 215–225 nm). To qualitatively confirm the binding of MMIPs to SNCA, MMIPs were prepared with encapsulated quantum dots (QDs). These MMIPs were incubated with SNCA at concentrations ranging from 1–30 µg/mL; then, the aggregated MMIPs were stained with rabbit anti-SNCA primary antibodies, followed by Alexa- fluor labeled anti-rabbit secondary antibodies. The aggregates were examined in a fluorescence microscope, as shown in Figure 4c. The red labeling of QD@MMIP was highly visible in all of these samples, and anti-SNCA staining (green) was colocalized with the red QD labeling. These representative images were obtained to demonstrate the binding of SNCA to MMIP aggregates by an additional method, and to verify that the QD labeling of MMIPs overlaps with SNCA immunostaining.

**Figure 5:**
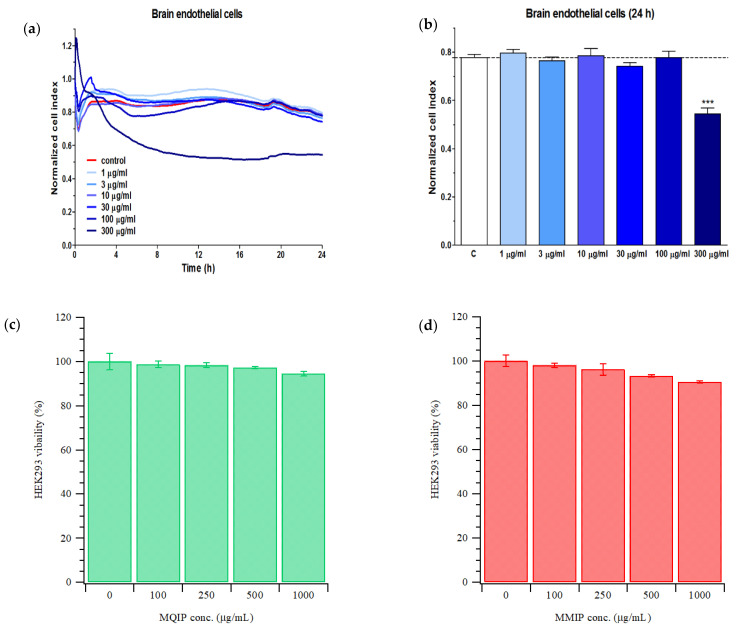
The effect of different concentrations of MMIPs on the (**a**) cell viability kinetics and (**b**) cell viability at 24-h time points of endothelial cells. Cultured primary endothelial cells were treated with various concentrations of MMIPs (1 µM to 300 µM) for 24 h. Control group received culture medium. Mean ± SD, *n* = 4–12, ANOVA, Dunnett’s multiple comparison test, *** *p* < 0.001 compared to the control group. Cytotoxicity of various concentrations of (**c**) magnetic CRISPR/Cas9 peptide-imprinted polymers (MQIPs) and (**d**) magnetic SNCA peptide-imprinted polymers (MMIPs) on HEK293T cells.

The cellular viability of brain endothelial cells treated with various concentrations of MMIPs is presented in Figure 5. MMIPs in concentrations up to 100 μg/mL did not damage endothelial cells after the 24-h treatment (Figure 5a). However, the highest concentration, 300 μg/mL, did cause significant cell toxicity compared to the control group (Figure 5b). Finally, the CRISPR/dCas9a system was used to activate the overexpression of SNCA in HEK293T cells, in order to create an in vitro model to test SNCA extraction. The details of the experiments can be found elsewhere [25]; they are also briefly described in the electronic Supplementary Material (ESM) S2 of the present study. We employed magnetic CRISPR/Cas9 peptide-imprinted polymers (MQIPs) to introduce CRISPR/dCas9 RNPs into the HEK293T cells. We also tested the cytotoxicity of the MQIPs (for SNCA induction, see Figure 5c) and the cytotoxicity of MMIPs (for SNCA extraction, see Figure 5d), both on HEK293T cells. Even at high concentrations, cellular viability remained >90%.

**Figure 6:**
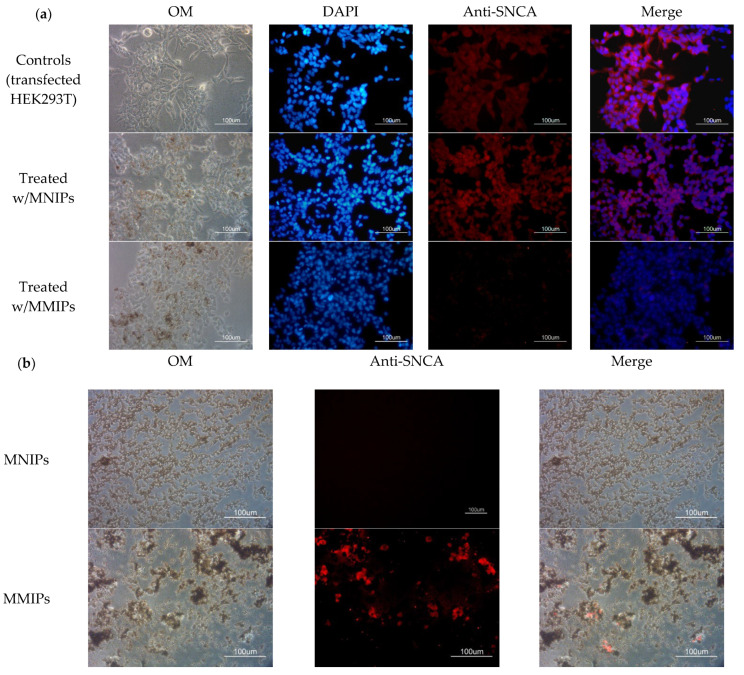
(**a**) Optical, nuclear (DAPI) staining, and immunostaining images of anti-SNCA proteins and merge images of HEK293T cells treated with MNIPs or MMIPs. (**b**) Optical and immunostaining images of anti-SNCA protein and merge images of MNIPs or MMIPs.

The top row of Figure 6a displays the activated expression of SNCA in HEK293T cells on the third day of treatment with CRISPR/dCas9, using immunostaining with anti-SNCA. Clearly, SNCA (red) was located in the transfected HEK293T cells. The second and third rows of Figure 6a present cells after extraction with MNIPs and MMIPs. When extracted with MMIPs, the red staining of SNCA is considerably reduced, compared with transfected cells in the first row; there may also be a very slight reduction in SNCA with MNIPs, owing to non-specific binding. Figure 6b displays optical and immunostaining (anti-SNCA) images of MNIPs and MMIPs following extraction; the MMIPs, but not the MNIPs, show clear red staining for SNCA, thus confirming a specific extraction of SNCA by the imprinted particles. These results demonstrate that the MMIPs can be used as extraction agents in the removal of aggregated proteins (such as SNCA) from cells. 

## 4. Conclusions

The recognition of proteins is of interest not only for diagnosis [26,27,28], but also for the targeting of therapeutics [30,31]. Epitope imprinting of SNCA has been demonstrated for the sensing of SNCA in Parkinson’s brain organoid culture medium [27], raising the possibility that such imprinted particles might also be useful for SNCA extraction. In this work, MMIPs were prepared using a 15-mer peptide template (P5 peptide) from the N-terminal alpha-helical amphipathic part of SNCA that contains the KTKEGV consensus sequence. These MMIPs not only bind the target peptide, but also SNCA in its monomeric form. We have previously shown that MMIPs can also bind SNCA filamentous aggregates [27], though likely with lower affinity (Figure S5D in [27]). Importantly, high concentrations (35–70 µM) of α-synuclein lead to fibrillization [32], while low concentrations (<0.4 μM) lead to the disaggregation of fibrils [33]. Thus, MIPs recognizing even monomeric SNCA may have therapeutic potential, as well as diagnostic potential. As an example of the latter in [27], MIPs, as part of electrochemical sensors, were able to detect SNCA from culture medium of Parkinson’s brain organoids, indicating the diagnostic potential of SNCA MIPs. 

To examine therapeutic potential, we used CRISPR technology [25] to construct an in vitro disease model for the aggregation of SNCA, for the purposes of studying the extraction of SNCA. Peptide-imprinted polymeric nanoparticles recognizing SNCA were synthesized, and magnetic nanoparticles were incorporated to facilitate separation and extraction processes. The ability of these particles to bind and extract SNCA from the in vitro model system was confirmed, while non-imprinted control particles had little effect. While targeting and clearance issues remain to be addressed, recent studies and trials have explored the use of antibodies for the reduction of neurodegenerative aggregates ([3] and references therein). It is noteworthy that clinical trials for the antibody-mediated clearance of β-amyloid [34] are being undertaken without targeting; thus, although therapeutic potential would benefit from targeting, it is not an essential prerequisite. In addition, a recent study has suggested a role for the liver in the clearance of SNCA in Parkinson’s disease brain pathology [35]. This result is encouraging for the possible future therapeutic application of MMIPs for the extraction or isolation of undesirable protein aggregates. Moreover, the use of magnetic nanoparticles as a tool to enhance the delivery of therapeutic molecules to the blood–brain barrier is particularly promising [36]. 

## Data Availability

The authors confirm that the data supporting the findings of this study are available within the article and its supplementary materials.

## Supplementary Materials

The following supporting information can be downloaded at: https://www.mdpi.com/article/10.3390/cells11162584/s1. Supplementary File S1: Materials and Methods [10,25,37,38] (Figure S1: CD spectra of P5–P7 peptides. The peptide concentration was 0.1 mg/mL; Figure S2: ITC of P5 MMIPs titrated with continuously 2 μL of P5 at 0.1 mg/mL.); Supplementary File S2: CRISPR.

## Abbreviations

AFM	atomic force microscope
BET	Brunauer–Emmett–Teller
CD	circular dichroism
CRISPR	clustered regularly interspaced short palindromic repeat
DAPI	4′,6-diamidino-2-phenylindole
dCas9a	denatured CRISPR-associated protein 9 with activator
DLS	dynamic light scattering
EVAL	poly(ethylene-*co*-vinyl alcohol),
HEK293T	human embryonic kidney 293 T cell line
HPLC	high-performance liquid chromatography
ITC	isothermal titration calorimetry
MMIPs	magnetic molecularly imprinted composite nanoparticles
MNIPs	magnetic non-imprinted polymeric particles
MNPs	magnetic nanoparticles
P5	YVGSKTKEGVVHGVA
P6	YVGSKTKEGVVHGVT
P7	YVGSKTKKGVVHGVT
PD	Parkinson’s disease
QDs	quantum dots
SNCA	α-synuclein
SQUID	superconducting quantum interference device
