# Supplementary material for: Cellular Therapy Using Epitope-Imprinted Composite Nanoparticles to Remove α-Synuclein from an In Vitro Model
Source: Cells. 2022 Aug 19;11(16):2584. doi: 10.3390/cells11162584 (PMC9406856; doi:10.3390/cells11162584)
Supplement: Supplementary file 1 [file cells-11-02584-s001.zip › Supplementary File S1-Materials & MEthods.pdf]

Electronic supplementary material:

Cellular Therapy Using Epitope-Imprinted Composite Nanoparticles to Remove α-Synuclein from an *in Vitro* Model

Mei-Hwa Lee[1], James L. Thomas[2], Yuan-Pin Shih[3], Jin-An Li[3], Chien-Yu Lin[3], Tooru Ooya[4,5], Lilla Barna[6,7], Mária Mészáros[6], András Harazin[6], Gergő Porkoláb[6,7], Szilvia Veszelka[6], Maria A. Deli[6,], Jeng-Shiung Jan[8,*], and Hung-Yin Lin[3,*]*

[1]Department of Materials Science and Engineering, I-Shou University, Kaohsiung 84001, Taiwan

[2]Department of Physics and Astronomy, University of New Mexico, Albuquerque, NM 87131, USA

[3]Department of Chemical and Materials Engineering, National University of Kaohsiung, Kaohsiung 81148, Taiwan

[4]Graduate School of Engineering, Department of Chemical Science and Engineering, Kobe University, Kobe 657-8501, Japan

[5] Center for Advanced Medical Engineering Research & Development (CAMED), Kobe University, Kobe 657-8501, Japan

[6]Institute of Biophysics, Biological Research Centre, H-6726 Szeged, Hungary

[7]Doctoral School in Biology, University of Szeged, H-6720 Szeged, Hungary

[8]Department of Chemical Engineering, National Cheng Kung University, Tainan, 70101, Taiwan

* To whom correspondence should be addressed:
Department of Chemical and Materials Engineering,
National University of Kaohsiung (NUK),
700, Kaohsiung University Rd.,
Nan-Tzu District, Kaohsiung 811, Taiwan
Tel: (O) +886(7)591-9455; (M) +886(912)178-751
E-Mail: linhy@ntu.edu.tw or linhy@caa.columbia.edu, deli.maria@brc.hu; jsjan@ncku.edu.tw

S1. Experimental

S1.1 Reagents and chemicals.

Poly(ethylene-*co*-vinyl alcohol), EVAL, with ethylene 27, 32, 38 and 44 mol%, iron (III) chloride 6-hydrate (97%) and 3-(4,5-dimethyl-2-thiazolyl)-2,5-diphenyl-2H-tetrazolium bromide (MTT) were from Sigma-Aldrich Chemical Company (St. Louis, MO). Iron (II) sulphate 7-hydrate (99.0%) from Panreac (Barcelona, Spain), acetic acid (ACS grade) from J. T. Baker (Phillipsburg, NJ) and sodium hydroxide from Mallinckrodt Chemical Inc. (St. Louis, MO). Three peptides, YVGSKTKEGVVHGVA (P5), YVGSKTKEGVVHGVT (P6), and YVGSKTKKGVVHGVT (P7), were ordered from Yao-Hong Biotechnology Inc. (HPLC grade, New Taipei City, Taiwan). Quantum dot kits (QDot 565 IKT) were purchased from Invitrogen Co. (Carlsbad, CA). All chemicals were used as received unless otherwise mentioned.

S1.2 Synthesis of magnetic optical peptide-imprinted chitosan nanoparticles

The synthesis of magnetic peptide-imprinted EVAL composite nanoparticles was modified from our previous work [1]. The synthesis steps included the preparation of magnetic nanoparticles: Magnetic nanoparticles (MNPs), synthesized by co-precipitation of a mixture of iron (III) chloride 6-hydrate and iron (II) sulfate 7-hydrate by sodium hydroxide, were repeatedly washed while adsorbed on a magnetic plate. The magnetic nanoparticles were

washed three times with deionized water, and then dispersed using an ultrasound bath for 30 s. Then, the phase transformation molecular imprinting of EVAL on the MNPs and QDs: Peptide was dissolved in DMSO at concentrations of 100 μg/mL. 250 μL DMSO/ EVAL solution was added into the same volume of peptide solution to form a clear EVAL solution, and 10 mg of the magnetic nanoparticles and 1 μL of QDs were then added. The EVAL was precipitated by dispersing 0.5 mL EVAL solution into 10 mL deionized water; then template was removed by washing in 10 mL deionized water 15 mins (3X), separating the MMIPs magnetically after each washing. The magnetic non-imprinted polymers (MNIPs) were prepared identically, but without peptide addition.

S1.3 Characterization of the MMIPs and MNIPs

The nanoparticles prepared as described in the previous section were monitored by a DLS particle sizer (90Plus, Brookhaven Instruments Co., New York) before and after peptide removal and rebinding. In the latter case, the magnetic nanoparticles were resuspended before dynamic light scattering measurements. Atomic force microscopy (AFM, Dimension Icon, Bruker) was used to examine particle morphologies; the resolutions in X-Y axis are <0.15nm and Z axis is < 30pm (closed loop). The particles were dispersed in phosphate buffered saline (PBS) and scanned by tapping mode with phase images.

The adsorption of peptide on nanoparticles was examined with a HPLC system. A peptide solution (1 mg/mL) in water was diluted to various concentrations for calibration. The sample (20 μL) was eluted with a mobile phase composed of 0.1% trifluoroacetic acid (TFA) in water (solvent A) and 0.1% TFA in acetonitrile (solvent B). The gradient profile of peptide p5 was as follows: 0–0.1 min, 12% A, 88% B; 0.1–25 min, 37% A, 63% B; 25.01–30 min, 100% A. The flow rate and detection wavelength were set to 1.0 mL/min and 220 nm, respectively. The calibrated retention times for peptides p5, p6 and p7 were 10.4, 9.6 and 7.0 min, respectively.

Nitrogen adsorption measurements were performed with a NOVA 1000e, and Brunauer–Emmett–Teller (BET) analysis was performed with the Autosorb program (Quantachrome Instruments, Florida). The magnetization of magnetic, peptide-imprinted polymers nanoparticles before and after template removal was monitored with a magnetic property measurement MPMS XL-7 system (Quantum Design, San Diego, CA) at 298 K in ±15000 Gauss.

The secondary conformation of P5-P7 peptides were analyzed in a 0.1 mm quartz cell by a JASCO J-815 circular dichroism (CD) spectrometer. The peptides were dissolved in PBS (pH 7.4, 0.15 M). Isothermal titration calorimetry (ITC) experiments between MMIPs and P5 peptide were carried out using a MicroCal iTC200 microcalorimeter (Malvern Instruments Ltd.). MMIPs were suspended in distilled water (33 μg/ml, 6.6 μM assuming 0.198 mmol/g

binding sites). The P5 peptide was also dissolved in distilled water (1.0 mg/ml, 0.66 mM). The MMIPs suspension was placed in the calorimeter cell. The titration syringe was loaded with the P5 peptide solution. The titrations were carried out with 18 injections of 2 μL each with time intervals of 4 second. The solution was stirred at 650 rpm. Titrations were carried out at a cell temperature of 25 °C and with a reference power of 5 μcal/s. ITC data analyses were carried out in Origin 7 SR 2 (OriginLab Corp).

S1.4 Cytotoxicity of MMIPs with primary rat brain endothelial cells

The isolation of primary rat brain endothelial cells was done as described in our previous study [2]. After isolation, cells were seeded onto collagen type IV and fibronectin coated (100 μg/mL each) Petri dishes (100 mm; Corning, USA). Cells were maintained in DMEM-F12 supplemented with 15% plasma-derived bovine serum (PDS; First Link, UK), 5 μg/mL insulin, 5 μg/mL transferrin, 5 ng/mL sodium selenite (Pan Biotech, Germany), 10 mM HEPES, 1 ng/mL basic fibroblast growth factor, 100 μg/mL heparin and 50 μg/mL gentamycin. During the first 3 days of culture the capillary endothelial cells were kept in culture medium containing 3 μg/mL puromycin to eliminate P-glycoprotein negative cell types [2], and cells were used at the first passage for experiments.

The kinetics of endothelial cells' reaction to nanoparticles were monitored by impedance

measurement (RTCA-SP instrument; Agilent, Santa Clara, CA, USA). Impedance measurement is a label-free, real time and noninvasive method that correlates linearly with adherence and growth of cells. After background measurements, cells were seeded at a density of 6×10^3 cells/well in collagen type IV and fibronectin coated (100 µg/mL each) 96-well plates with integrated gold electrodes (E-plate 96, Agilent). Cells were cultured in a CO_2 incubator at 37 ºC and monitored. At the beginning of the plateau phase of growth, 3-5 days after seeding, cells were treated with magnetic nanoparticles at 1, 3, 10, 30, 100, 300 µg/mL concentrations for 24 hours. Cell index was defined as R_n-R_b at each time point of measurement, where R_n is the cell-electrode impedance of the well when it contains cells and R_b is the background impedance of the well with the medium alone. Cell index values reflect cell number and viability [3].

S1.5 Activation of SNCA gene in HEK 293T cells by the delivery of CRISPR/dCas9-VPR: SNCA Ribonucleoproteins

Gene activation in cells by the delivery of CRISPR/dCas9-VPR: ribonucleoproteins (RNPs) was recently reported [4]. The 5'- 3' sequence of crRNA (GACCAGAAGGGGCCCAAGAG) used to activate transcription of SNCA and tracrRNA were obtained from Dharmacon, Inc. (Lafayette, CO). Equal amounts of 5µL crRNAs and tracrRNA at 5 nM were mixed at room temperature for 30 min to form gRNAs. Ten microliters of the extracted dCas9-VPR at 100

μg/mL was loaded with SNCA gRNAs for 30 min at room temperature to form dCas9a SNCA RNP. This RNP was then immobilized on 1.0 mg of magnetic Cas9 peptide-imprinted (MQIPs) nanoparticles. HEK 293T cells were incubated with 100 μg/mL nanoparticles for 2 days to activate the SNCA gene. These cells were then washed and grown in medium for 5 additional days (with two additional changes of media), and then cells were examined using immunohistochemistry (fluorescence) to study SNCA expression.

S1.6 SNCA immunolabeling

Human recombinant SNCA (AnaSpec Inc, Fremont, CA, USA) was dissolved in PBS at a concentration of 500 μg/mL. MMIPs labeled with quantum dots (QD; 100 μg/mL) were incubated with 1, 3, 10, 30 μg/mL SNCA for 30 min at room temperature. After washing three times with PBS, MMIPs were incubated with anti-SNCA primary antibody (1:1000, rabbit polyclonal, recombinant anti-SNCA antibody MJFR1, #ab138501; Abcam, UK) for 30 min at room temperature. After another 3X wash with PBS, the particles were incubated with secondary antibody anti-rabbit-Alexa Fluor 488 (1:1000, A11029, Thermo Fisher Scientific Inc., USA). After a final 3X PBS, the particles were mounted in Fluoromount-G (Southern Biotech, USA) and examined with a Leica SP5 (Leica Microsystems GmbH, Germany) confocal laser scanning microscope.

S1.7 Statistics

Data are presented as means ± SD. Values were compared using one-way ANOVA following Dunnett multiple comparison posttest (GraphPad Prism 5.0; GraphPad Software, USA). Changes were considered statistically significant at $P < 0.05$. The number of parallel samples was 4-12.

Reference:

[1] M.-H. Lee, J.L. Thomas, C.-L. Liao, S. Jurcevic, T. Crnogorac-Jurcevic, H.-Y. Lin, Epitope recognition of peptide-imprinted polymers for Regenerating protein 1 (REG1), Separation and Purification Technology, 192 (2018) 213-219.

[2] S. Veszelka, A. Tóth, F.R. Walter, A.E. Tóth, I. Gróf, M. Mészáros, A. Bocsik, É. Hellinger, M. Vastag, G. Rákhely, Comparison of a rat primary cell-based blood-brain barrier model with epithelial and brain endothelial cell lines: gene expression and drug transport, Frontiers in molecular neuroscience, 11 (2018) 166.

[3] A. Harazin, A. Bocsik, L. Barna, A. Kincses, J. Váradi, F. Fenyvesi, V. Tubak, M.A. Deli, M. Vecsernyés, Protection of cultured brain endothelial cells from cytokine-induced damage by α-melanocyte stimulating hormone, PeerJ, 6 (2018) e4774.

[4] M.-H. Lee, C.-C. Lin, J.L. Thomas, J.-A. Li, H.-Y. Lin, Cellular reprogramming with multigene activation by the delivery of CRISPR/dCas9 ribonucleoproteins via magnetic peptide-imprinted chitosan nanoparticles, Materials Today Bio, 9 (2021) 100091.

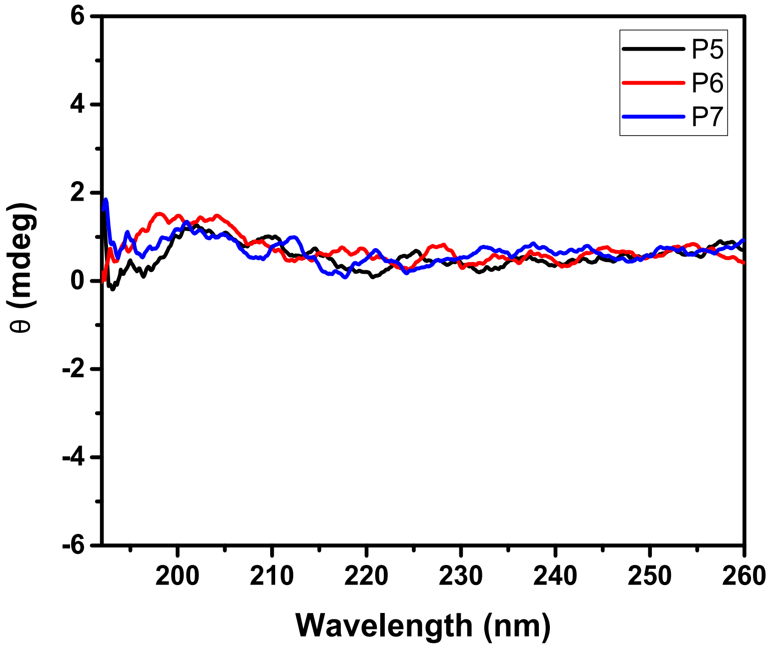

Figure S1. CD spectra of p5-p7 peptides. The peptide concentration was 0.1 mg/mL.

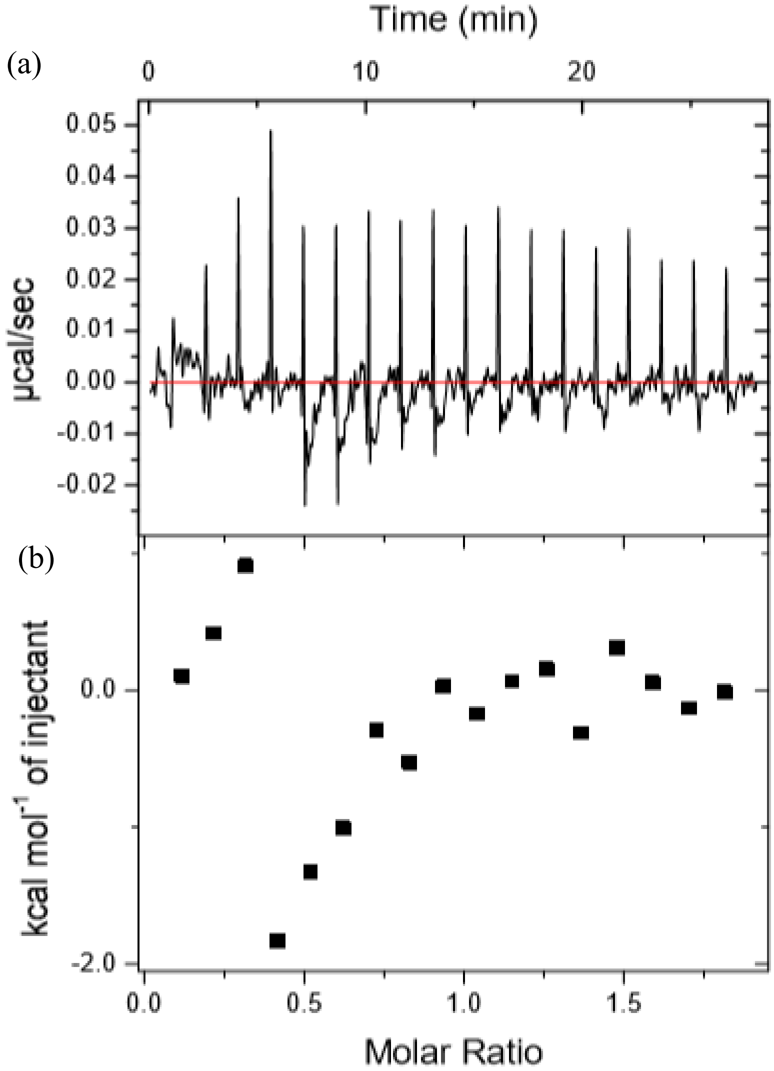

Figure S2. ITC of p5 MMIPs titrated with continuously 2 µL of P5 at 0.1 mg/mL.
