# Supplementary material for: Cellular Therapy Using Epitope-Imprinted Composite Nanoparticles to Remove α-Synuclein from an In Vitro Model
Source: Cells. 2022 Aug 19;11(16):2584. doi: 10.3390/cells11162584 (PMC9406856; doi:10.3390/cells11162584)
Supplement: Supplementary file 1 [file cells-11-02584-s001.zip › Supplementary File S2-CRISPR.pdf]

Electronic supplementary material:

Cellular Therapy Using Epitope-Imprinted Composite Nanoparticles to Remove α-Synuclein from an *in Vitro* Model

Mei-Hwa Lee[1], James L. Thomas[2], Yuan-Pin Shih[3], Jin-An Li[3], Chien-Yu Lin[3], Tooru Ooya[4,5], Lilla Barna[6,7], Mária Mészáros[6], András Harazin[6], Gergő Porkoláb[6,7], Szilvia Veszelka[6], Maria A. Deli[6,], Jeng-Shiung Jan[8,*], and Hung-Yin Lin[3,*]*

[1]Department of Materials Science and Engineering, I-Shou University, Kaohsiung 84001, Taiwan

[2]Department of Physics and Astronomy, University of New Mexico, Albuquerque, NM 87131, USA

[3]Department of Chemical and Materials Engineering, National University of Kaohsiung, Kaohsiung 81148, Taiwan

[4]Graduate School of Engineering, Department of Chemical Science and Engineering, Kobe University, Kobe 657-8501, Japan

[5] Center for Advanced Medical Engineering Research & Development (CAMED), Kobe University, Kobe 657-8501, Japan

[6]Institute of Biophysics, Biological Research Centre, H-6726 Szeged, Hungary

[7]Doctoral School in Biology, University of Szeged, H-6720 Szeged, Hungary

[8]Department of Chemical Engineering, National Cheng Kung University, Tainan, 70101, Taiwan

* To whom correspondence should be addressed:
Department of Chemical and Materials Engineering,
National University of Kaohsiung (NUK),
700, Kaohsiung University Rd.,
Nan-Tzu District, Kaohsiung 811, Taiwan
Tel: (O) +886(7)591-9455; (M) +886(912)178-751
E-Mail: linhy@ntu.edu.tw or linhy@caa.columbia.edu, deli.maria@brc.hu; jsjan@ncku.edu.tw

S2. Experimental

S2.1 Reagents and chemicals.

Chitosan (C3646) was purchased from Sigma-Aldrich Chemical Company (St. Louis, MO), acetic acid (ACS grade) from J. T. Baker (Phillipsburg, NJ) and sodium hydroxide from Mallinckrodt Chemical Inc. (St. Louis, MO). Peptide of Cas9 protein in the sequence of QLFVEQHKHYLDE was purchased from Yao-Hong Biotechnology Inc. (HPLC grade, New Taipei City, Taiwan). Human embryonic kidney cells (HEK 293T) were purchased from ATCC (American Type Culture Collection).

S2.2 Preparation of magnetic peptide-imprinted chitosan composite nanoparticles and their delivery of RNPs HEK 293T cells

The preparation of MQIPs is previously reported; briefly, magnetic nanoparticles (MNPs) were added to a chitosan solution (0.01 wt% chitosan and 0.01 wt % acetic acid in deionized (DI) water) to an MNP concentration of 0.2 mg/mL. The chitosan/MNPs solution was then mixed with 0.1 g/mL of peptide for the preparation of magnetic peptide imprinted MQIPs. The template was removed by washing with 10 mL deionized (DI) water for 1 hr and then separating the MQIPs using the field from a magnetic plate, both two times.

S2.3 Immunohistochemistry of SNCA proteins

HEK293T (2x10^4) cells were seeded in 24-well cell plates and kept at 37ºC in 5% CO_2 for 24 hr. Cells were then washed with 400 µL PBS in each well, and fixed in 3.7 % formaldehyde in PBS (pH 7.4) for 10 min at room temperature. After washes with 350 µL PBS in each well, cells were permeabilized with 1 % Triton X-100 for 5 min at room temperature, and then washed with 350 µL/well PBS, followed by blocking of nonspecific binding by washing in PBS supplemented with 5% BSA for 60 mins. Finally, 350 µL PBS was added in each well and cells were incubated overnight at 4 °C with 1:250 rabbit anti-SNCA antibody (Sino Biological, #101282-T02) to BSA. The cells were then washed 3 times in PBS for 5 min each and labeled with 300 µL/well secondary antibody for 1 h at room temperature. After another wash in 250 µL PBS at room temperature, the cells were co-stained with the nuclear dye DAPI for 15 mins (Sigma). Finally, the cells were washed with PBS and examined with an inverted fluorescence microscope (CKX41, Olympus, Melville, NY).
